# Protein arginine methyltransferase 5 is essential for virulence in *Toxoplasma gondii*

**DOI:** 10.1186/s13071-026-07473-3

**Published:** 2026-06-02

**Authors:** Limei Xu, Huiru Liang, Shengchen Bai, Ruochen Xu, Bin Liu, Xiaohong Xu, Xinrong Xu, Min Liu

**Affiliations:** 1 Institute of Tropical Medicine, Department of Pathogen Biology, School of Public Health, Southern Medical University, Guangdong Provincial Key Laboratory of Tropical Disease Research, Key Laboratory of Prevention and Control for Emerging Infectious Diseases of Guangdong Higher Institutes, Key Laboratory of Infectious Diseases Research in South China, Ministry of Education, Guangzhou, 510515 Guangdong China; 2 Department of Traditional Chinese Medicine, The Second Affiliated Hospital of Guangzhou Medical University, Guangzhou, 510515 Guangdong China

**Keywords:** *Toxoplasma gondii*, Protein arginine methyltransferase 5, Virulence, Bradyzoite differentiation, Gene expression

## Abstract

**Background:**

Protein arginine methyltransferase 5 (PRMT5) is a key enzyme responsible for catalyzing symmetric dimethylarginine (SDMA) modifications and plays crucial roles in epigenetic regulation, transcription, and cell cycle progression in eukaryotes. Although our previous study determined the expression and cellular localization of PRMT5 in tachyzoites and bradyzoites, and confirmed its type II PRMT activity, its functional significance in *Toxoplasma gondii* remains entirely uncharacterized.

**Methods:**

This study aimed to explore the biological functions of PRMT5 in *T. gondii*. The *prmt5* gene was disrupted in the type I RH strain using the clustered regularly interspaced short palindromic repeats (CRISPR) Cas9 system. The biological roles of PRMT5 were evaluated via multiple functional assays, including plaque formation, intracellular proliferation, host cell invasion, virulence, and tachyzoite to bradyzoite conversion assays. RNA sequencing was further performed to profile transcriptomic alterations induced by *prmt5* disruption.

**Results:**

Phenotypic characterization revealed that the ∆*prmt5* strain exhibited reduced symmetric dimethylarginine (SDMA) levels as well as severe defects in plaque formation, invasion, intracellular replication, and bradyzoite differentiation. Accordingly, the virulence of the ∆*prmt5* strain was dramatically attenuated, as all infected BALB/c mice survived over a 10-day period, in stark contrast to the 100% mortality observed in the wild-type control group within 10 days. RNA-sequencing analysis uncovered the molecular basis for these phenotypes, demonstrating that *prmt5* disruption leads to global transcriptional dysregulation. Specifically, we identified a significant downregulation of genes associated with motor protein function and fatty acid metabolism pathways.

**Conclusions:**

Our research has demonstrated that PRMT5 plays a critical role in the proliferation, survival, pathogenicity, and regulation of gene expression in *Toxoplasma gondii*.

**Graphical Abstract:**

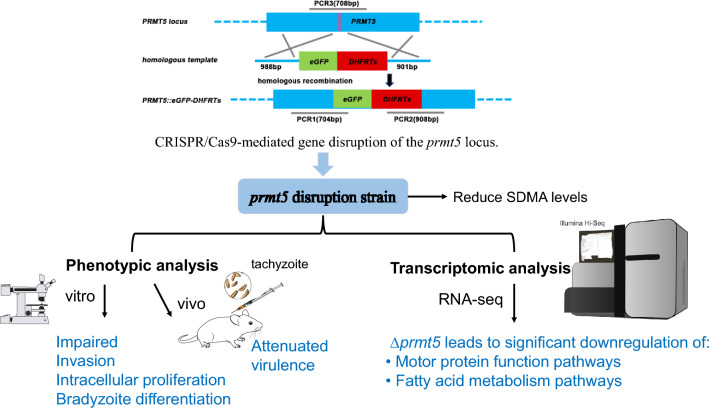

**Supplementary Information:**

The online version contains supplementary material available at 10.1186/s13071-026-07473-3.

## Background

*Toxoplasma gondii *(*T. gondii*) is an opportunistic pathogenic protozoan that can invade almost all nucleated cells and widely infect warm-blooded hosts including humans [1]. *Toxoplasma gondii* infection in immunocompetent individuals are generally asymptomatic, however, the parasite forms latent bradyzoites-containing cysts in brain and muscle tissues for many years [2, 3]. When hosts become immunosuppressed, the parasite can cause severe infections including Toxoplasma encephalitis, pneumonia, eye disease, and even death. Primary infection in pregnant women can lead to adverse pregnancy outcomes such as abortion, preterm labor, fetus death, and deformity, resulting in public health burden [4, 5]. One-third of the world’s population is estimated to be infected with *T. gondii*. Severe outbreaks of toxoplasmosis have been reported in several countries, and these infections have led to significant public health concerns [6, 7].

The complex life cycle of *T. gondii* encompasses multiple distinct stages, each characterized by unique biological and morphological features that contribute to specific pathological outcomes and clinical manifestations. Post-translational modifications (PTMs) regulate numerous cellular processes and play a role in various aspects of cell biology. In addition, PTMs can be induced or removed on changes in cellular environment and state. Thus, PTMs are likely to be key regulators of developmental transitions, biology, and pathogenesis of apicomplexan parasites [8]. PTMs refers to the modification of proteins at one or more sites that can determine the conformation, subcellular localization, type of interacting proteins, and protein stability and activity. Common PTMs include phosphorylation, acetylation, ubiquitination, methylation, and hydroxylation [9]. Arginine methylation is an abundant post-translational modification that occurs in mammalian cells and is mediated by protein arginine methyltransferases (PRMTs) [10, 11]. In humans, PRMTs catalyze three types of methylation: type I (PRMT1–4, PRMT6, and PRMT8), which forms mono methylarginines (MMA) and asymmetric dimethylarginines (ADMA); type II (PRMT5 and PRMT9), which forms MMA and symmetric dimethylarginines (SDMA); and type III (PRMT7), which forms only MMA [12, 13]. These processes are involved in many important cellular processes, including epigenetic regulation, RNA processing, DNA damage response, hormone-receptor signaling, and cell proliferation and differentiation [10, 14]. PRMT enzymes are conserved in many kingdoms of life, with extended sets of PRMTs present in protozoa compared with other simple eukaryotes [15]. The assortment of PRMTs in each protozoan organism suggests that PRMTs play unique roles in the biology of different parasites [16, 17]. PRMT5 is a typical type II methyltransferase and widely distributed and highly conserved [18]. PRMT5 contributes to the histone code by catalyzing the symmetrical dimethylation of histone H3R8 and H4R3 (H3R8me2s and H4R3me2s), a modification typically associated with transcriptional repression [19–21]. Despite its well-characterized roles in model systems, the functions of PRMT5 and other arginine methyltransferases remain poorly understood in *T. gondii*.

Gene expression in *T. gondii* is dynamically regulated throughout its life cycle—during replication, stage differentiation, and host–parasite interactions. These life cycles indeed necessitate stringent regulation of gene expression. While many phenotypic differences in *T. gondii* are genetically encoded, epigenetic mechanisms may play a critical role in the parasite’s developmental programming and its adaptation to environmental fluctuations. Notably, *T. gondii* lacks detectable DNA cytosine methylation, making histone PTMs a major regulatory layer for chromatin remodeling and gene control [22, 23].

Our previous study [24] has shown that *T. gondii* arginine methyltransferase 5 (TgPRMT5) has intrinsic type II PRMT activity, which is a PRMT5 homolog and may participate in gene repression in *T. gondii*. Furthermore, there is significant difference in the expression localization of TgPRMT5 in different stages of parasite, with the protein localization in cytoplasm in tachyzoites; however, it mainly localized in the nucleus in bradyzoites. Therefore, TgPRMT5 is proposed to play an important role of virulence and tachyzoite–bradyzoite conversion in *T. gondii*.

Given the important role of PRMT5 in *T. gondii,* it is necessary to explore its role in the infection process and the contribution to the parasite growth and virulence of *T. gondii*. In this study, we used the clustered regularly interspaced short palindromic repeats (CRISPR)-Cas9 technique to edit *prmt5* gene in the *T. gondii* RH strain to construct disruption strain and examined the function in the pathogenicity by a series of phenotypic experiments in vitro and in vivo.

## Methods

### Mice and parasite strains

Specific pathogen free (SPF) BALB/c mice (8 weeks old, weighing 16–18 g, male) were used in this study, which were purchased from Medical Laboratory Animal Center of Guangdong Province. The study was performed in a standard SPF environment in accordance with the animal welfare and related ethical requirements of the Animal Ethics Committee of Guangdong Provincial Medical Animal Center (GY-2024-765). Before conducting formal experiments, all mice were acclimatized for 3 days under appropriate temperature and environmental conditions. The *T. gondii* strains used in this study, including wild-type (WT) RH strain, RH-GFP strain expressing green fluorescent protein (GFP) [25], and *prmt5* disruption strain (RHΔ*prmt5* strain), were maintained in vitro in human foreskin fibroblast (HFF) cells and were propagated in Dulbecco’s modified Eagle’s medium (DMEM; Life Technologies, Inc., Rockville, MD, USA) supplemented with 1% fetal bovine serum (FBS), penicillin, and streptomycin.

### Construction of *prmt5* disruption strain

The CRISPR/Cas9 system was adopted to disrupt *prmt5* gene with eGFP-DHFRTs integrated as a selection marker. The sgPRMT5 was designed to target the upstream region of the S-adenosylmethionine (SAM)-dependent methyltransferase active domain of TgPRMT5. We used NEB Q5 Site-Directed Mutagenesis Kit to replace the UPRT targeting gRNA with prmt5 specific gRNA in the pSAG1-CAS9-sgUPRT plasmid (pSAG1::CAS9-U6::sgPRMT5). Briefly, the 5′ and 3′ homology arms of sgRNA (∼1 kb) were amplified from the genomic DNA of the RH strain and cloned into the pBluescript II SK (-) vector along with the enhanced green fluorescent protein (eGFP) and pyrimethamine selection cassette DHFRTs (pBlu-5HR-eGFP-DHFRTs-3HR-down). The pSAG1::CAS9-U6::sgPRMT5 plasmid and the TgPRMT5 homology fragment vector pBlu-5HR-eGFP-DHFRTs-3HR-down were mixed at a 5:1 ratio and electroporated into freshly collected tachyzoites of *T. gondii* RH strain; 3 μM pyrimethamine was added 48 h post-electroporation for selection. Single clones were isolated by limiting dilution and then screened by fluorescence and verified by polymerase chain reaction (PCR) to confirm the disruption of *prmt5* and integration of the eGFP-DHFRTs cassette. The primers used in this study are listed in Supplementary Table 1.

### Western blot analysis for detection of SDMA levels and GFP protein

Tachyzoites of RH-WT and RH∆*prmt5* strains were harvested by scraping the infected cells with a cell scraper and purified by passage through a 3 µm filter (Whatman, UK). The purified tachyzoites were washed twice with ice-cold phosphate-buffered saline (PBS) and pelleted by centrifugation. The resulting parasite pellet was lysed in radio immunoprecipitation assay (RIPA) buffer (Solarbio, China) supplemented with protease inhibitors. The mixture was thoroughly resuspended by pipetting and incubated on ice for 30 min. The lysate was then centrifuged at 12,000 g for 10 min at 4 °C, and the supernatant containing the total protein extract was collected and stored at −80 °C. Equal amounts of total protein (10 μg per lane) were separated by 12% sodium dodecyl sulfate polyacrylamide gel electrophoresis (SDS-PAGE). Following electrophoresis, proteins were transferred onto a PVDF membrane (BIO-RAD, USA) using a wet transfer system at a constant current of 400 mA for 100 min in an ice bath. The membrane was blocked with 5% bovine serum albumin (BSA, ACMEC, China) in tris-buffered saline with tween 20 (TBST) for 1 h at room temperature. The membrane was then incubated with rabbit anti-symmetric dimethylarginine (anti-SDMA) antibody (1:1000, Cell Signaling Technology) overnight at 4 °C.

GFP protein expression was detected using a rabbit anti-GFP monoclonal antibody (1:20,000, HuaBio), and *β*-tubulin was used as a loading control (1:500, a generous gift from Professor Hongjuan Peng). After washing with TBST, the membrane was incubated with an horseradish peroxidase (HRP)-conjugated goat anti-rabbit IgG secondary antibody (1:5000, ABclonal) for 1 h at room temperature. Following further washes, protein bands were visualized using anenhanced chemiluminescence (ECL) chemiluminescence substrate (Beyotime, China), and images were captured using a chemiluminescence imaging system.

### Effect of *prmt5* gene disruption on plaque formation in RH-WT strain

The in vitro growth rates of the RH-WT and RH∆*prmt5* strains were compared by counting and measuring the number and size of plaques formed on HFF cells monolayers. Briefly, HFF cells were cultured into 12-well plates for 24 h. Then, freshly purified parasites were collected and infected with HFF cells at a dose of 10,000 tachyzoites/well. Subsequently, the 12-well plates containing parasites were incubated at 37 °C in a humidified environment with 5% CO_2_ for 7 days. After incubation, the culture medium was discarded and infected cells were washed twice with PBS, and then fixed in 4% paraformaldehyde (4% PFA, Biosharp, China) for 30 min. Fixed cells were washed twice with PBS and stained with 0.1% crystal violet (Beyotime, China) for 30 min. After drying at room temperature naturally, the plaque formed by tachyzoites infection could be observed. The size and number of each plaque was determined by using an inverted microscope as previously described.

### Effect of *prmt5* gene disruption on invasion ability in RH-WT strain

To evaluate the invasion efficiency of RHΔ*prmt5* and RH-WT strains, HFF cells were cultured on glass coverslips in 12-well plates. The monolayers were infected with 4 × 10^5^ GFP-expressing tachyzoites per well and incubated at 37 °C with 5% CO_2_ for 4 h to allow parasite attachment and invasion. After incubation, extracellular parasites were labeled by immunostaining without permeabilization. Cells were fixed with 4% paraformaldehyde (PFA) for 20 min at room temperature, washed with PBS, and blocked with 3% BSA in PBS for 1 h. Without permeabilization, samples were incubated with mouse anti-*Toxoplasma* SAG1 antibody (1:500, Invitrogen, USA) for 2 h at room temperature to stain extracellular parasites. After washing with PBS, cells were incubated with iFluor™ 594-conjugated goat anti-mouse IgG (1:1000, Thermo Fisher Scientific, USA) for 2 h at room temperature. Nuclei were stained with DAPI (Solarbio, China) for 15 min. Coverslips were examined under an Eclipse TS100 upright fluorescence microscope (Nikon, Japan). Total parasites were identified by GFP fluorescence, while extracellular parasites were identified by red fluorescence. Invasion rate was calculated as the number of intracellular tachyzoites/total tachyzoites. Data were obtained from three independent experiments.

### Effect of *prmt5* gene disruption on intracellular proliferation ability in RH strain

To assess the intracellular replication capacity of the RHΔ*prmt5* and RH-WT strains, HFF cells were cultured on glass coverslips in 12-well plates until confluent. The monolayers were infected with 2 × 10^5^ GFP-expressing tachyzoites per well. After 2 h of incubation to allow for parasite invasion, extracellular parasites were removed by washing three times with PBS, and fresh medium was added. Infected cells were then incubated at 37 °C with 5% CO_2_ for 24 h. At 24 h post-infection, cells were fixed with 4% PFA in PBS for 30 min at room temperature, washed with PBS, and stained with a DAPI solution for 15 min to visualize the nuclei. Coverslips were then air-dried and examined under an Eclipse TS100 fluorescence microscope. The number of tachyzoites per parasitophorous vacuole (PV) was counted manually for at least 100 randomly selected vacuoles per sample. Vacuoles containing 1, 2, 4, 8, 16, or more parasites were scored, and the percentage of vacuoles in each category was calculated. Data were obtained from three independent experiments.

### Effect of *prmt5* gene disruption on acute infection

To examine the effect of *prmt5* disruption on acute infection, BALB/c mice were randomly divided into two groups (ten mice per group) and fresh egressed tachyzoites of the RH-WT strain and RHΔ*prmt5* strains were injected intraperitoneally (I.P.) into mice at a dose of 100 tachyzoites. Subsequently, the infected mice were monitored at least twice daily for clinical symptoms of toxoplasmosis and mortality status was recorded for 30 days. On day 8 post-infection with *T. gondii*, mice were euthanized and liver and spleen tissues were aseptically harvested. Approximately 10 mg of each tissue was homogenized to generate a single-cell suspension. The suspension was then centrifuged at 10,000 × g for 1 min, and the supernatant was carefully removed. DNA was subsequently isolated using the Genomic DNA Extraction Kit (TIANGEN, China), and the concentration and purity of each DNA sample were determined spectrophotometrically. The *T. gondii* burden in the liver and spleen of mice from both groups was subsequently quantified using quantitative real-time PCR (qPCR), using the B1 gene as the target gene.

### Bradyzoite differentiation in vitro

To explore the role of *prmt5* in the bradyzoite differentiation, as previously described [26], approximately 1 × 10^5^ tachyzoites of RH-WT and RHΔ*prmt5* were inoculated into T25 culture flasks containing HFF cells and allowed to invade host cells for 4 h at 37 °C under 5% CO_2_. Following invasion, the culture medium was removed and replaced with a maintenance medium composed of DMEM supplemented with 1% FBS, 20 mM HEPES buffer (4-(2-hydroxyethyl)-1-piperazineethanesulfonic acid), and 1% penicillin–streptomycin, adjusted to pH 8.2. Cultures were maintained under standard incubation conditions at 37 °C, with daily medium changes to ensure pH stability. Total RNA was harvested on days 2, 4, 5, and 6 post-invasion, while an undifferentiated control group was collected at day 0. Subsequently, qPCR was performed to relatively quantify the expression levels of SAG1 and BAG1 in both parasite strains across all timepoints, using *β*-tubulin as the reference gene. The primers used for qPCR are listed in the Supplementary Table 2.

### Tachyzoites collection and RNA extraction

HFFs were infected with either the RH-WT or RH∆*prmt5* strain at a multiplicity of infection (MOI) of 2 (two tachyzoites per HFF). At 2 h post-infection, both the control group (infected with the RH-WT strain) and the experimental group (infected with the RH∆*prmt5* strain) were supplemented with 4 mL of fresh culture medium and incubated for an additional 48 h. Each group consisted of three biological replicates. After the 48-h incubation period, tachyzoites were collected and stored at −80 °C for RNA extraction and RNA sequencing (RNA-seq).

Total RNA was individually extracted from each sample using TRIzol reagent (Invitrogen, USA), following the manufacturer’s instructions. All extracted RNA was treated with RNase-Free water (sheng gong, China) to remove any residual genomic DNA. The integrity and purity of extracted RNA were assessed using the Nanodrop 2000 spectrophotometer (Thermo Scientific™, USA) and the Agilent 2100 Bioanalyzer system (Agilent Technologies, USA), respectively.

### RNA-seq and reads mapping

Equal amounts of total RNA from the RH-WT and RH∆*prmt5* strains were used and poly(A) RNA was isolated using the Oligo (dT) enrichment method. The RNA was fragmented randomly into segments ranging from 200 to 500 bp, followed by cDNA synthesis via reverse transcription. The ends of the cDNA fragments were repaired and phosphorylated at the 5’ end, generating a protruding ‘A’ nucleotide at the 3’ end. Adapters with a complementary ‘T’ overhang were then ligated to these ends. The resulting ligation products were amplified by PCR and enriched into sequencing libraries using specific primers. Paired-ended sequencing was subsequently performed on the Illumina platform to generate the raw sequencing data.

Raw FASTQ sequences were treated with Trimmomatic tools (v0.36) using the following options: :TRAILING:20, SLIDINGWINDOW:4:15 MINLEN:52 to remove trailing sequences below a Phred quality score of 20 and to achieve uniform sequence lengths for downstream clustering processes. Subsequently, sequencing quality was assessed to obtain high-quality data (clean data). Concurrently, HISAT2 software was employed to align the sequencing data against the *T. gondii* genome. HTSeq (v0.12.4) was used to count the reads numbers mapped to each gene. The RNA-seq library construction and read alignment were performed by Ruibo Biological Co., Ltd.

### Analysis of differentially expressed genes (DEGs)

On the basis of the mapped reads with the reference genome, FPKM was used to count the expression level of each gene. Fragments per kilobase of exon per million mapped fragments (FPKM) refers to the number of fragments per thousand bases in length from a certain gene. This method takes into account the influence of sequencing depth and gene length on the count of fragments.

Gene expression differences were analyzed using DESeq2 software, which is based on the negative binomial generalize linear model. Gene expression with log_2_foldchange ≥ 1 or ≤   −1, and adjusted *P*-value < 0.05 was considered as differentially expressed. Kyoto Encyclopedia of Genes and Genomes (KEGG, http://www.genome.jp/) and Gene Ontology (GO, http://www.geneontology.org/) were used for gene functional annotation, pathway annotation, and gene enrichment analyses, respectively. The GO enrichment analysis results were categorized by biological process, cellular component, and molecular function.

### Verification of RNA-seq results by qPCR

The RNA-seq results were verified using qPCR. The expression levels of 15 DEGs were determined by qPCR using the same RNA samples that were sequenced, with *β*-tubulin as the reference gene. The RNA samples were reverse-transcribed into cDNA using the Trans Script® Uni All-in-One First-Strand cDNA Synthesis Super Mix for qPCR Reverse Transcription Kit (Quanshi Jin, China).

Quantification by qPCR was performed using the SYBR® Green Pro Taq HS Premix Kit (Accurate Biology, China) in the QuantStudio™ 6 Flex Real-Time PCR System (Thermo Fisher Scientific, USA). The 10-μL system contained 3.6 μL of RNase-free water, 5 μL of SYBR Green Pro Taq HS Premix, 0.4 μL of primers per pair, and 1μL of cDNA template. The selected genes were analyzed in triplicate. The amplification conditions are as follows: 95 °C for 30 s, followed by 30 s at 95 °C, then an extension for 30 s at 60 °C, repeated 40 times. Relative quantification was performed using the 2^−(ΔΔCT)^ method. The primers used for qPCR are listed in the Supplementary Table 2.

### Statistical analysis

All quantitative data were obtained from three triplicate experiments. Statistical significance was determined using GraphPad Prism 10.0 by two-tailed unpaired *t*-test or log-rank test (survival analysis), with *P* < 0.05 considered significant. For RNA-seq, differentially expressed genes were identified using DESeq2 with a Benjamini–Hochberg adjusted *P*-value < 0.05 and |log2(foldchange)|> 1.

## Results

### Construction of *prmt5* deficient mutant strain by CRISPR-Cas9

**Fig 1.**
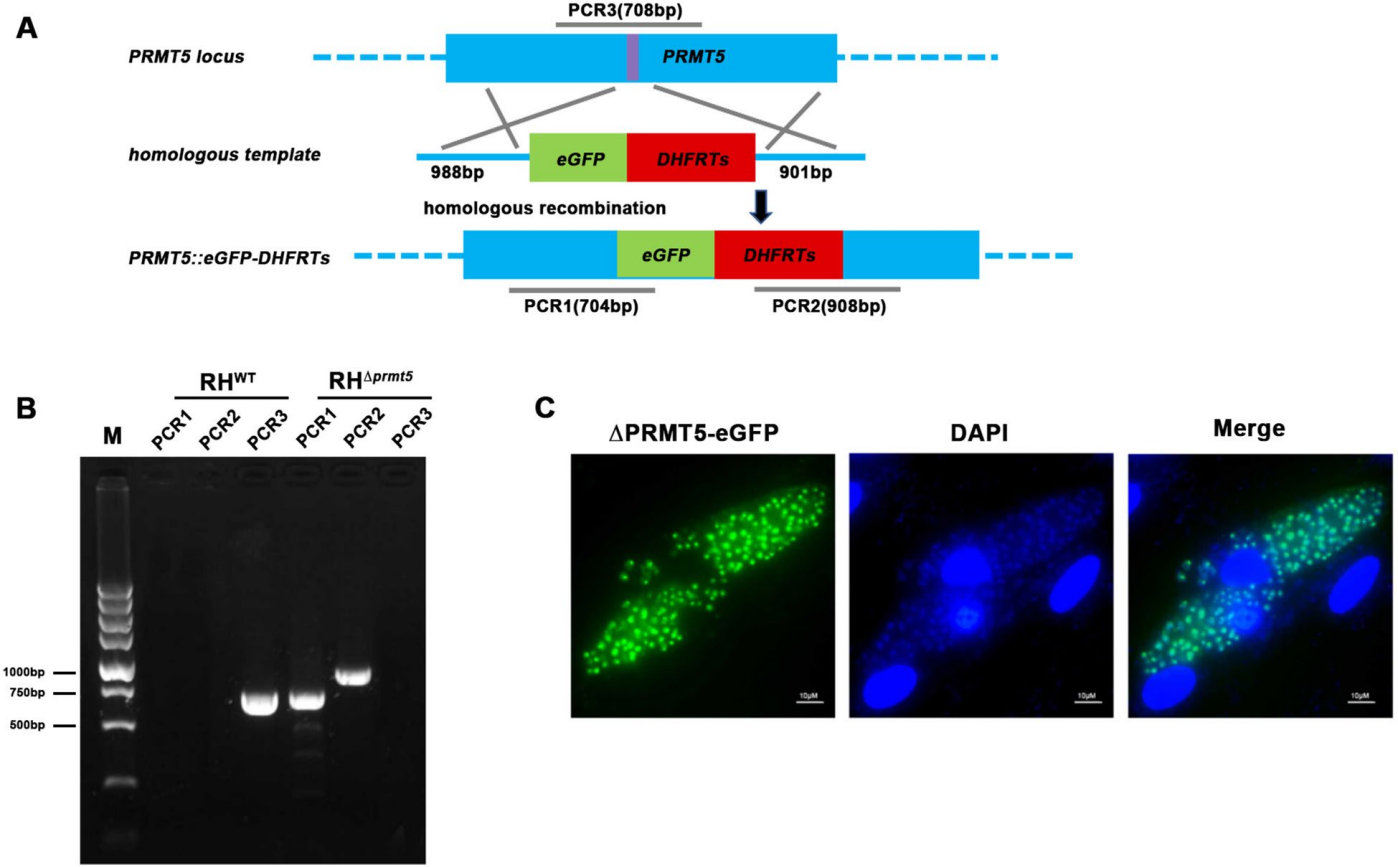
CRISPR/Cas9-mediated gene disruption of the *prmt5* locus. **A** Schematic representation of the CRISPR/Cas9 strategy for *prmt5* inactivation via insertional fusion of eGFP and pyrimethamine-resistant DHFRTs. **B** PCR validation of homologous integration and gene disruption in a representative clone compared with the RH parental strain tachyzoites. **C** Fluorescence microscopy revealed that eGFP was successfully fused to the residual fragment of *prmt5* and localized to the nucleus of *T. gondii*

**Fig 2.**
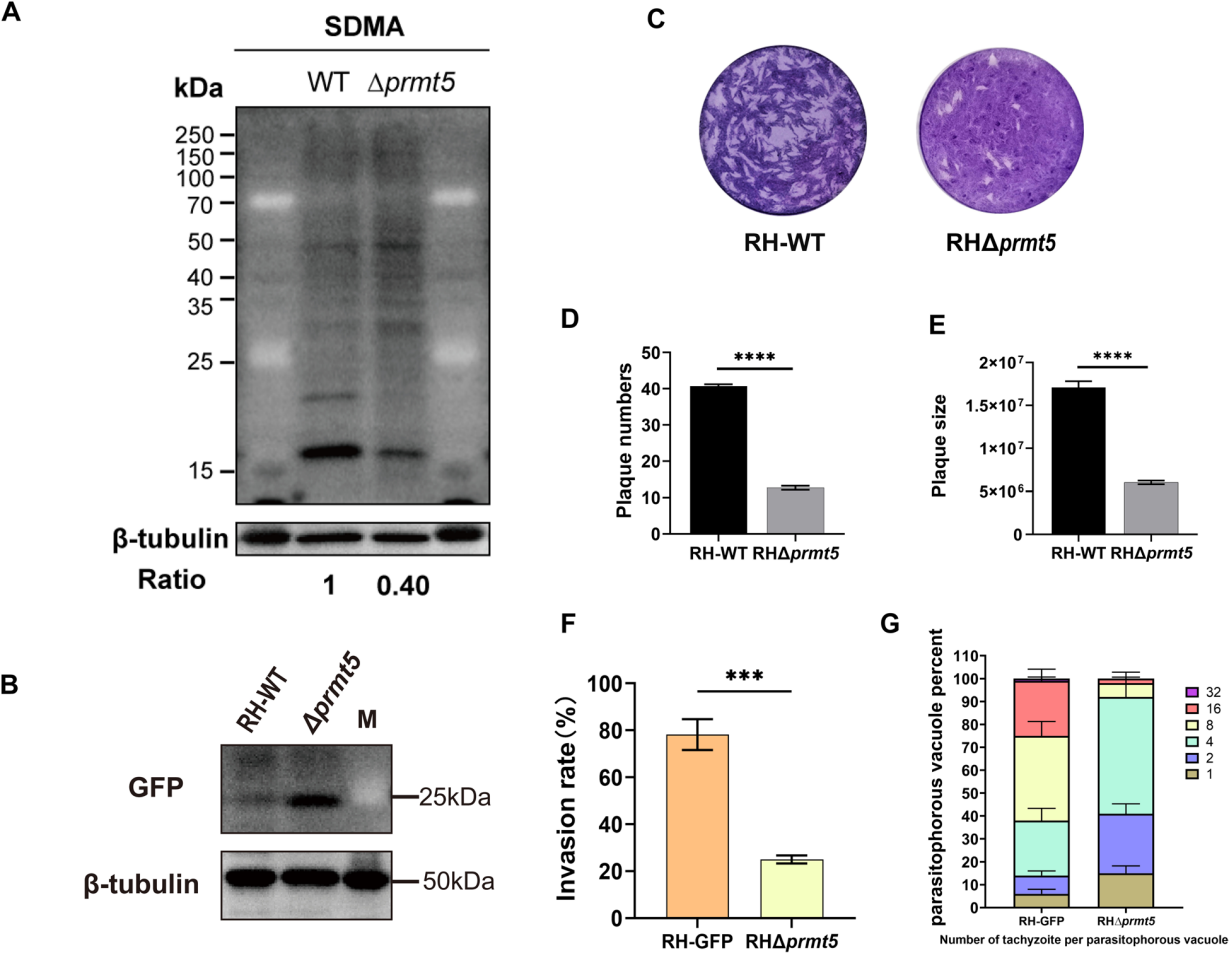
**A** SDMA levels in RH-WT and RH∆*prmt5* strains detected by western blot (two-tailed unpaired *t*-test, ****P < 0.0001). **B** The expression of GFP protein in RH∆*prmt5* was detected. **C** Representative photographs of plaques observed in HFFs infected by the RH-WT strains and RHΔ*prmt5*. **D** The number of plaques produced by RHΔ*prmt5*, showed a significant reduction compared with the RH-WT strain (Two-tailed unpaired *t*-test, ****P < 0.0001). **E** The size of plaques produced by RHΔ*prmt5*, showed a significant reduction compared with the RH-WT strain (two-tailed unpaired *t*-test, ****P < 0.0001). **F** Invasion assay of RHΔ*prmt5* and the RH-WT strains (two-tailed unpaired *t*-test, ***P < 0.001). **G** Proliferation assay of RHΔ*prmt5* and the RH-WT strains. All images are representative of results from three independent experiments

The CRISPR/Cas9 system was used to disrupt the *prmt5* gene in the RH-WT strain. The coding region of the gene was successfully replaced with the 5HR-eGFP-DHFRTs-3HR fragment (Fig. 1A). Single clones were generated via drug selection and limiting dilution and validated by PCR3. A ∼700 bp fragment was amplified by PCR3 in the RH-WT strain, whereas no amplification was observed in the RHΔ*prmt5* strain. Successful integration of the eGFP-DHFRTs fragment was further confirmed by PCR1 and PCR2. Specifically, a ∼700 bp fragment was amplified by PCR1, and a ∼900 bp fragment was amplified by PCR2 in the RHΔ*prmt5* strain, whereas neither band was detected in the WT strain (Fig. 1B). Fluorescence microscopy revealed that eGFP was fused to the residual *prmt5* fragment and localized to the nucleus of *T. gondii* (Fig. 1C). GFP protein expression in ∆*prmt5* was also successfully detected using Western blot (Fig. 2B). These results showed that CRISPR/Cas9-mediated homologous recombination enabled the successful generation of *prmt5* deficient mutant strain.

### Disruption of *prmt5* reduces SDMA levels in *T. gondii*

To determine whether TgPRMT5 deficiency impacts global symmetric arginine dimethylation in *T. gondii*, western blot analysis was conducted on total protein extracts from the RH-WT strain and the RH∆*prmt5* strain using an anti-SDMA antibody. As presented in Fig. 2A, abundant SDMA signals were predominantly detected at a molecular weight of 15–25 kDa, consistent with the canonical molecular size of core histones. Notably, the RHΔ*prmt5* strain displayed a pronounced reduction in SDMA signal intensity within this range relative to the wild-type control (*P* < 0.0001). Meanwhile, the weak residual SDMA signals in the RHΔ*prmt5* strain imply the presence of alternative type II PRMT activity in *T. gondii*, or a compensatory mechanism that partially rescues TgPRMT5 functional loss under physiological conditions.

### Disruption of *prmt5* gene affects the growth of parasite

To assess the impact of disrupting *prmt5* gene on the survival of RH-WT strain, plaque assays were performed on HFF cell monolayers in 12-well plates and the plaque number and size were analyzed. As shown in Fig. 2C–E, RHΔ*prmt5* strain exhibited a significant reduction in plaque sizes and numbers (*P* < 0.0001), indicating that *prmt5* gene disruption partially affects the *T. gondii* lytic cycle.

### Disruption of* prmt5* gene inhibits the invasion and intracellular replication of the RH-WT strain

RH∆*prmt5* and RH-WT strains tachyzoites were added to 12-well plates containing HFF cells and incubated in a 37 °C incubator for 4 h to assess tachyzoites invasion capacity. The results are presented in the Fig. 2F. The invasion rate of the RH∆*prmt5* strain was significantly lower than that of the RH-WT strain, indicating that the disruption of the *prmt5* gene hindered the invasion of *T. gondii*.

Intracellular proliferation was assessed by observing the number of tachyzoites in the vacuoles. HFFs were infected with tachyzoites of RHΔ*prmt5* and the RH-WT strains and the parasite replication rate was monitored by counting the number of tachyzoites per PV in at least 100 PVs. The number of tachyzoites in the PVs are shown in Fig. 2G. It was shown that the RH-WT strain mostly contained 4, 8, or 16 tachyzoites in PV, whereas the RH∆*prmt5* strain mostly contained 2 or 4 tachyzoites. Eight tachyzoites were relatively rare. The result suggests that RHΔ*prmt5* exhibited a significant decrease in the parasite intracellular proliferation.

### Disruption of *prmt5* gene attenuate the virulence of parasite

**Fig 3.**
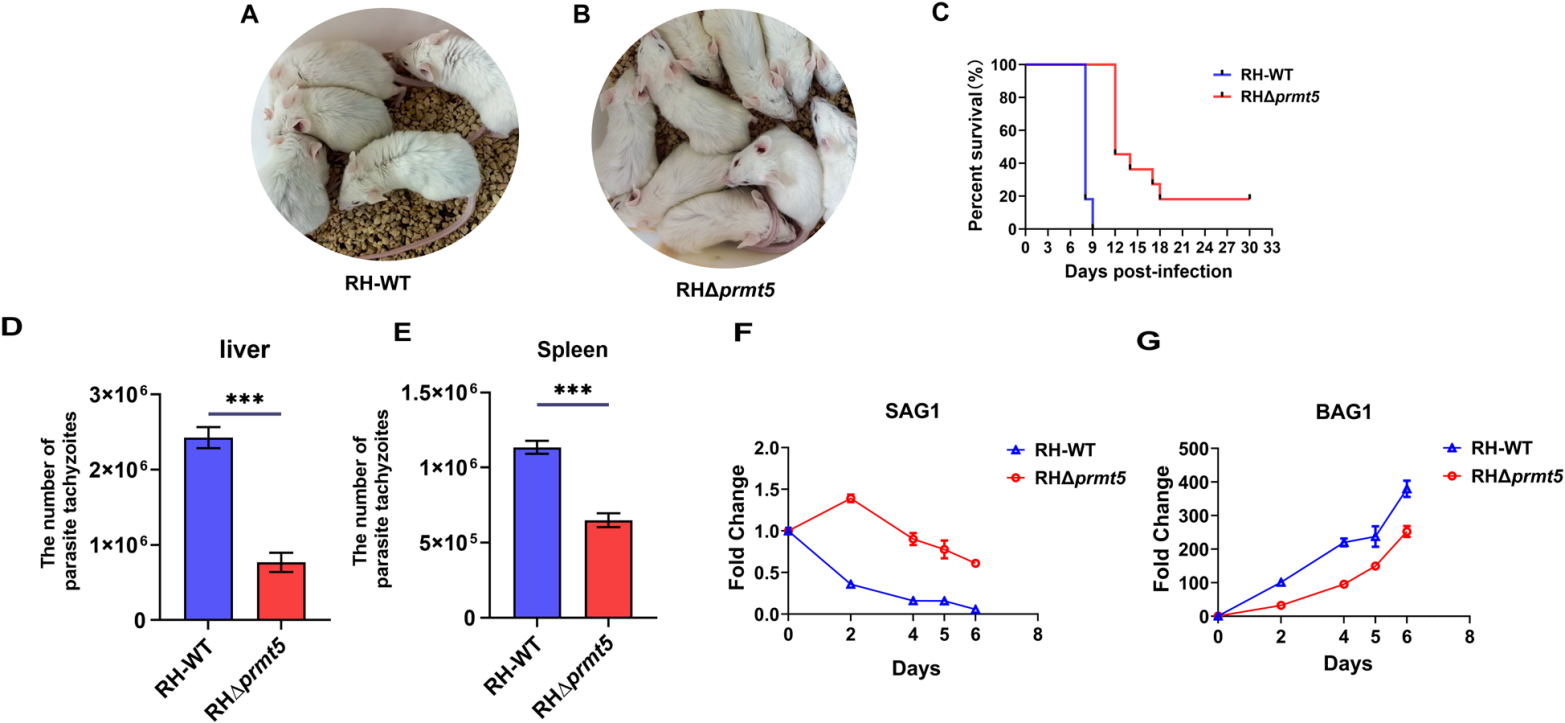
Virulence experiments of RH-WT and RH∆*prmt5* strains. **A** Mice in the RH-WT strain group began to show symptoms on day 6. **B** Mice in the RH∆*prmt5* strain group began to show symptoms on day 10. **C** Survival of BABL/c mice infected with RHΔ*prmt5* and the RH-WT strains (log-rank (Mantel–Cox) test, *P* < 0.0001). **D**, **E** The burden on the liver and spleen caused by RH-WT and RH∆*prmt5* strain infections in mice (two-tailed unpaired *t*-test, ***P < 0.001). **F**, **G** The expression levels of the SAG1 and BAG1 genes were analyzed on days 2, 4, 5, and 6 following bradyzoite differentiation induced by the RH-WT and RHΔ*prmt5* strains under alkaline conditions

To explore whether disruption of *prmt5* gene affects the virulence of RH-WT strain, the BALB/c mice (10 mice per group) were intraperitoneally (I.P.) injected with 100 freshly egressed tachyzoites of RHΔ*prmt5* or RH-WT strain. The condition of the mice was observed daily. It was found that on day 6 of the mice being infected with parasites, the RH-WT group showed poor conditions, such as bristling hair, arched back, and loss of appetite (Fig. 3A). However, the RH∆*prmt5* group of mice did not show the above symptoms until day 10 (Fig. 3B). The survival rates of all infected mice are shown in Fig. 3C. The results showed that during the 30-day observation period, all the mice infected with the RH-WT strain died within 9 days. However, among the mice infected with the RH∆*prmt5* strain, there were still two mice alive at the end of the observation period. Furthermore, mice infected with the RHΔ*prmt5* strain showed a significantly lower parasite burden in the liver and spleen than those infected with the RH-WT strain (Fig. 3D, E). These results confirm that the *prmt5* gene is essential for the full virulence of the RH-WT strain.

### Disruption of* prmt5* gene reduces bradyzoite differentiation

Previously, we investigated the subcellular localization of TgPRMT5. During the tachyzoite stage, TgPRMT5 is predominantly localized in the cytoplasm, whereas during the bradyzoite stage is primarily found in the nucleus [24]. To further examine whether TgPRMT5 influences bradyzoite differentiation, the pH of the culture medium was adjusted to 8.2 to induce bradyzoite differentiation in vitro. Our results showed that, compared with preinduction levels, the expression of the SAG1 gene decreased over time in both strains as induction progressed. In contrast, the expression of the BAG1 gene increased with prolonged induction; however, the BAG1 expression level in the RHΔ*prmt5* strain was significantly lower than that in the wild-type RH strain (Fig. 3F, G). Compared with the RH strain, disruption of the *prmt5* gene impaired the transition of tachyzoites to bradyzoites, resulting in delayed bradyzoite differentiation. The RHΔ*prmt5* strain exhibited consistently slower differentiation throughout the entire process. These findings demonstrate that TgPRMT5 acts as an essential regulator during the tachyzoite-to-bradyzoite developmental transition.

### Quality assessment of RNA-seq

The raw data were processed to eliminate unjoined sequences and low-quality reads, thereby generating clean data. After filtering out host-derived sequences, the remaining valid data were aligned to the reference genome using HISAT2 software. In the RH-WT group, the average mapping rate was 89.74%. For the RH∆*prmt5* group, the average alignment rate was 86.56%, showing a relatively uniform distribution across samples. These alignment results were in line with the expected outcomes. Detailed analysis results are presented in Supplementary Table 3.

In this study, we used the Pearson correlation coefficient (*r*) to measure the linear relationship between two variables, as illustrated in Fig. S1A. The results show that the *r* values among all biological duplicate samples are greater than 0.937 and approach the ideal state.

### Disruption of* prmt5* gene leads to extensive transcriptional regulatory disorders

We analyzed gene expression in HFF cells infected with *T. gondii* in the present or absence of the *prmt5* gene. A total of 8925 genes were included in the database. Of these, 8439 ± 42 were detected in the RH-WT strain and 8322 ± 16 in the RH∆*prmt5* strain. Fig. S1B presents the density distribution of gene expression abundance on the basis of the TPM (Transcipts per million) density distribution map. The RH∆*prmt5* strain exhibited a higher peak and increased density within the log2 (TPM) < 0 range. These findings preliminarily suggest that the disruption of *prmt5* gene results in the downregulation of the majority of genes.

**Fig 4.**
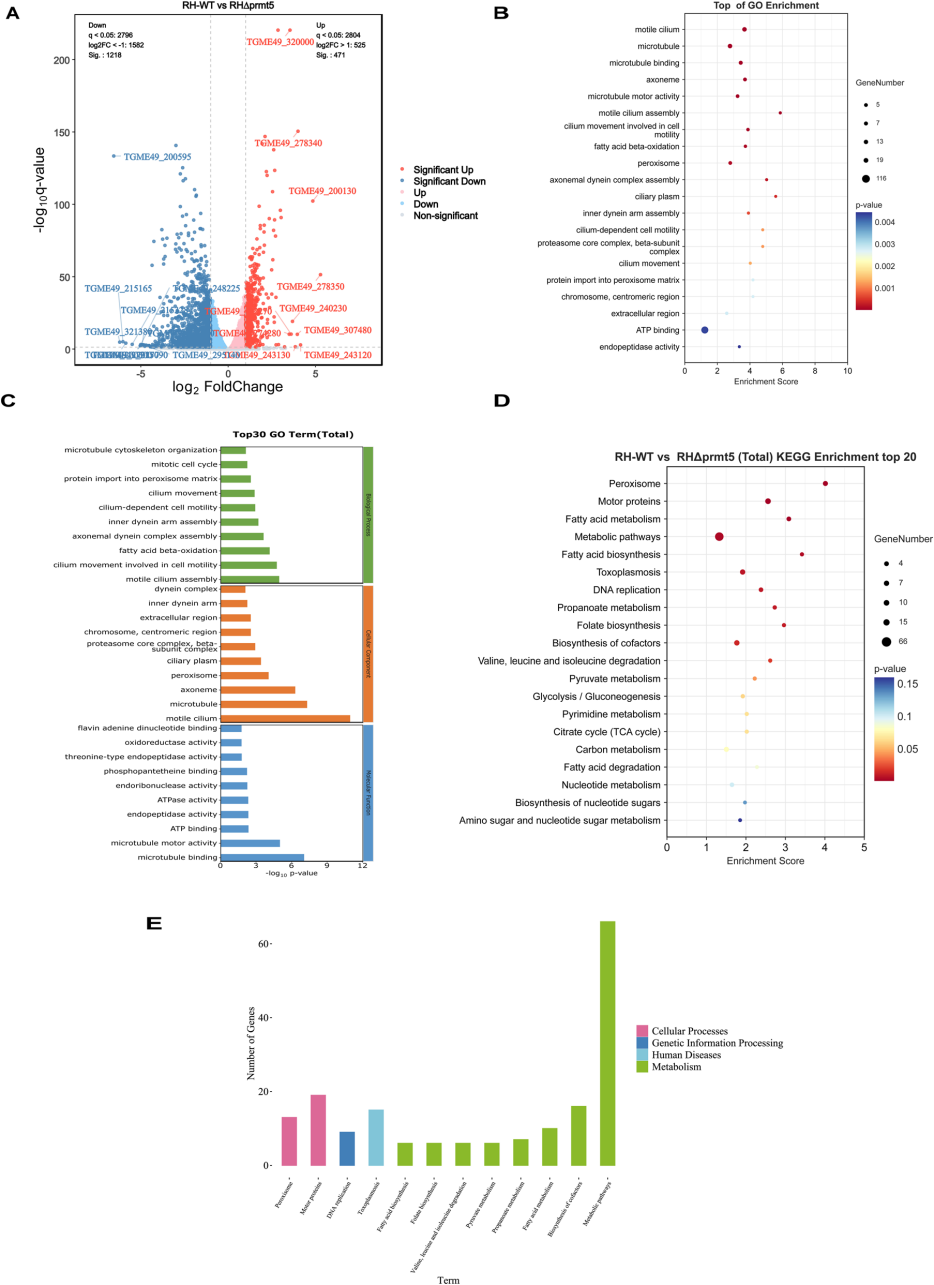
Bioinformatics analysis of the DEGs. **A**Volcano diagrams showing the DEGs between RH-WT strain and RH∆*prmt5* strain. The horizontal axis shows the multiples of changes in mRNA expression in different samples. The vertical axis shows the statistical significance of the differences in gene expression levels. Red dots indicate significant upregulation of mRNA, and blue dots indicate significant downregulation. **B** Bubble chart of GO enrichment distribution of DEGs. **C** Top 30 GO Term of DEGs. **D** Bubble chart of the KEGG pathway of DEGs. The horizontal axis of the figure shows the proportion of DEGs that are enriched among the background genes of the pathway. The vertical axis shows the pathway name. The size of the dots indicates the number of enriched DEGs, and the color represents the *P*-value (*P*-value < 0.05). **E** KEGG pathway gene enrichment distribution map of DEGs

We conducted an analysis of the DEGs in the RH-WT and RH∆*prmt5* strains using the DEseq2 method. DEGs were identified on the basis of the logarithmic fold change (log_2_foldchange ≥ 1 or ≤ −1) and an adjusted *P*-value < 0.05. Compared with the RH-WT strain, the expression levels of 1689 genes were found to being significantly altered in the RH∆*prmt5* strain. Specifically, 471 genes exhibited significant upregulation, while 1218 genes showed significant downregulation, as shown in Fig. 4A. The volcano plot provides a clear visualization of DEGs, highlighting the top ten genes exhibiting the most statistically significant upregulation and downregulation. Disruption of the *prmt5* gene results in a significant alteration in the gene expression profile of *T. gondii*, indicating that TgPRMT5 plays a critical role in regulating the transcriptional program of *T. gondii*.

### GO analysis of DEGs

GO analysis was performed to classify and characterize the DEGs between the RH-WT and RH∆*prmt5* strains. As shown in Fig. 4B, C, the GO enrichment bubble plot and the distribution of DEGs revealed that the functional categories of these DEGs primarily encompass three major aspects: biological processes, cellular components and molecular functions. Among these, the most significantly enriched terms were concentrated in three functional categories: microtubule motor activity, peroxisome, and fatty acid beta-oxidation. GO analysis of up- and downregulated genes was performed separately, and the results are presented in Fig. S2A, B. Downregulated genes were almost exclusively enriched in cytoskeleton-related functions, including cilium movement and assembly, microtubule, and microtubule motor activity, as well as in peroxisome and fatty acid oxidation-related functions. This suggests that TgPRMT5 regulates the expression of microtubule-based cytoskeleton-associated genes, thereby affecting the motility of *T. gondii*. Additionally, PRMT5 deficiency also inhibits fatty acid oxidation-related functions. In contrast, upregulated genes were involved in a variety of functions with a more dispersed distribution, primarily enriched in protein folding and degradation, translational elongation, and related processes.

### KEGG analysis of DEGs

The KEGG classification results demonstrate that DEGs are associated with four primary categories of KEGG pathways: cellular processes, genetic information processing, human diseases, and metabolism. KEGG pathway analysis revealed that the majority of DEGs of *T. gondii.* were mainly involved in metabolic pathways, peroxisomes, motor proteins, and other signaling pathways (Fig. 4D). As shown in Fig. 4E, 12 pathways exhibited the highest levels of enrichment. Among these, the top 6 pathways with the most significant enrichment of DEGs included metabolic pathways such as fatty acid metabolism and biosynthesis, toxoplasmosis, DNA replication, motor proteins, and peroxisomes. As shown in Fig. S2C, D, the downregulation of genes was significantly enriched in the dynein and metabolic pathways, particularly in the pathway of unsaturated fatty acid metabolism and peroxisome, and the upregulated genes are mainly concentrated in pathways such as proteasome, DNA replication and repair, toxoplasmosis, and fatty acid biosynthesis. These results indicate that TgPRMT5 plays a critical role not only in the regulation of gene expression, but also in the control of motility and metabolic processes in *T. gondii*. Such disruption results in substantial impairments in parasite motility and metabolic homeostasis.

### qPCR verification of DEGs

**Fig 5.**
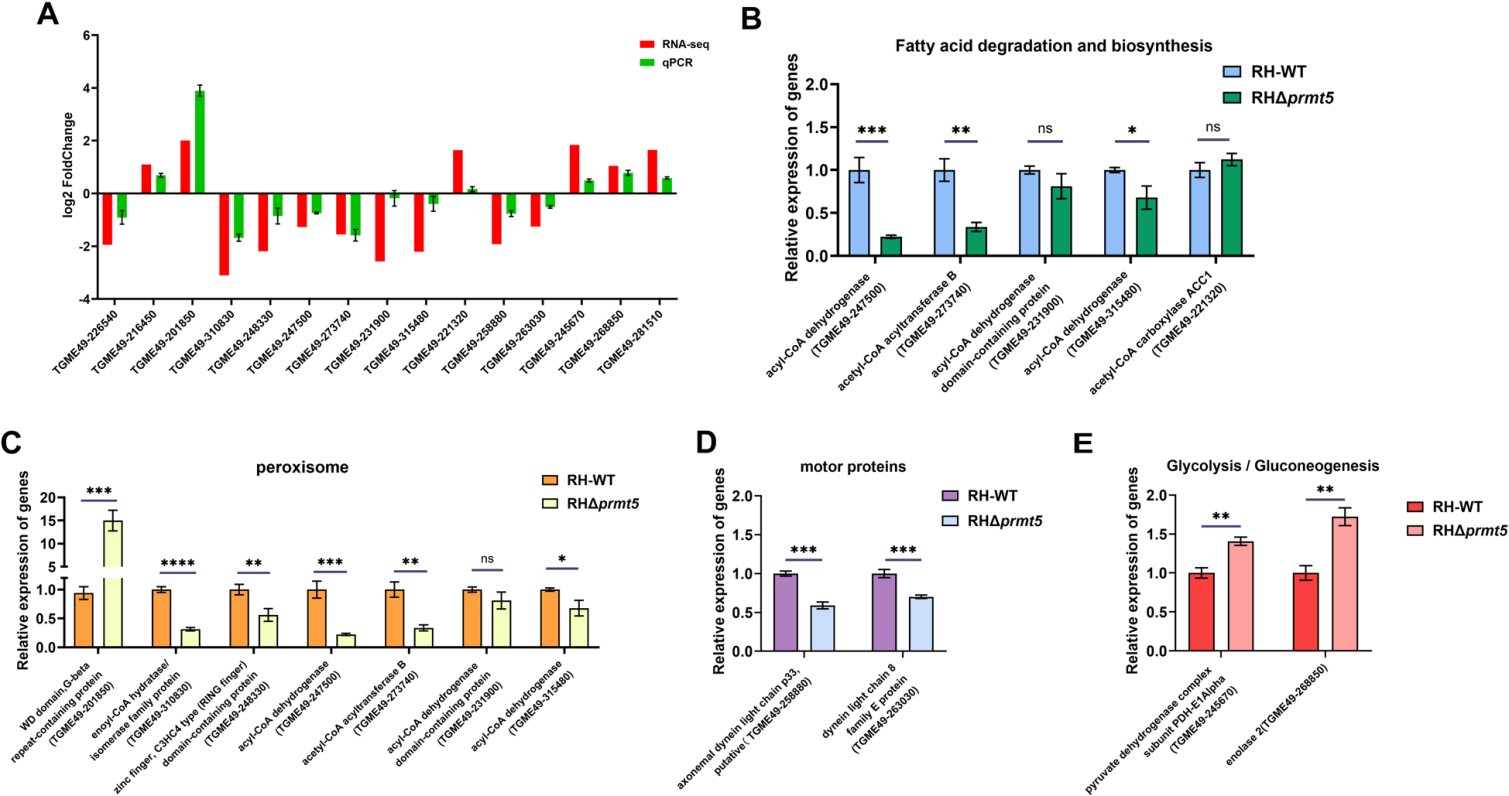
qPCR verification of DEGs. **A** Verification of the 15 DEGs by using qPCR. **B** The mRNA expression levels of genes in fatty acid metabolism were detected by qPCR. **C** The mRNA expression levels of genes in peroxisome were detected by qPCR. **D** The mRNA expression levels of genes in motor proteins were detected by qPCR. **E** The mRNA expression levels of genes in glycolysis/gluconeogenesis were detected by qPCR. The statistical significance of intergroup differences was assessed using a two-tailed unpaired *t*-test. (ns, *P* > 0.05; *P < 0.05; **P < 0.01; ***P < 0.001; ****P < 0.0001.)

To validate the reliability of the RNA-seq results and further substantiate the regulatory role of TgPRMT5 in metabolic and motility-associated pathways, we selected 15 representative DEGs from these pathways for qPCR validation. The selected genes encompass key components of significantly enriched pathways, including fatty acid metabolism (e.g., (acyl-CoA dehydrogenase, middle domain-containing protein, TGME49-247500), (acetyl-CoA acyltransferase B, TGME49-273740), (acyl-CoA dehydrogenase domain-containing protein, TGME49-231900), (acyl-CoA dehydrogenase, middle domain-containing protein, TGME49-315480), and (acetyl-CoA carboxylase ACC1, TGME49-221320)) (Fig. 5B) and motor protein-related processes (e.g., (axonemal dynein light chain p33, TGME49-258880), (dynein light chain 8 family E protein, TGME49-263030)) (Fig. 5D), as well as peroxisome (Fig. 5C), glycolysis/gluconeogenesis (Fig. 5E), DNA replication, and toxoplasmosis-related signaling pathways. The detailed information of the 15 DEGs can be found in Supplementary Table 4. Although the magnitude of change differs slightly from that in the RNA-seq data, the overall expression trends were consistent with the RNA-seq findings (Fig. 5A). Notably, several genes involved in fatty acid degradation, peroxisome, and motor protein functions were indeed downregulated due to *prmt5* gene disruption. These results not only corroborate the accuracy of the RNA-seq data, but also reinforce the role of TgPRMT5 as a critical transcriptional regulator in *T. gondii*.

## Discussion

This study demonstrates that TgPRMT5 plays multifaceted and essential roles in *T. gondii*, critically regulating tachyzoite invasion, intracellular replication, virulence, and stage conversion. Integrating comprehensive phenotypic characterization with transcriptomic profiling of ∆*prmt5* parasites, we provide evidence that TgPRMT5 is essential for maintaining the expression of genes associated with motor proteins, peroxisomes, and fatty acid metabolism pathways, with its deficiency leading to phenotypic abnormalities. These findings position PRMT5 as a key regulator that can coordinate both structural integrity and metabolic homeostasis required for critical life cycle processes in *T. gondii*.

The obvious defects in invading host cells and intracellular replication observed in the RH∆*prmt5* strain may be related to the downregulation of motor protein-related gene expression. GO and KEGG enrichment analyses revealed that the downregulated genes were almost all concentrated in processes related to microtubules, including cilium movement and assembly, components of the dynein complex, and microtubule motility activity. These transcriptional changes align with a growing body of research that indicates that arginine methylation directly regulates the cytoskeleton in apicomplexan parasites and related organisms. For example, in *Trypanosoma brucei*, α-tubulin is modified by SDMA, while *β*-tubulin and *ε*-tubulin are modified by MMA and SDMA, respectively [27]. This suggests that methylation may play a role in tubulin regulation, cytoskeleton integrity, and intracellular transport. In *T. gondii*, the glideosome is a large molecular complex composed of a motility complex located in the parasite’s plasma membrane and adhesion proteins, which plays a crucial role in the parasite’s movement and host cell invasion [28–30]. The motility complex consists of the *T. gondii* myosin A heavy chain and two associated light chains: TgMLC1 and either TgELC1 or TgELC2 [31, 32]. It is notable that the latest proteomics analysis suggests the motility complex of *T. gondii* (including MyOA, MLC1, ELC1, GAP45, and GAP50) is extensively modified by various PTMs such as palmitoylation, ubiquitination, phosphorylation, and methylation [8]. This indicates that this motility complex is the main target of multiple modifications and may achieve precise regulation of the parasite’s movement through the synergistic effect of these modifications. Additionally, a previous study reported that the ∆*prmt1* strain exhibited the loss of synchronous replication ability and abnormal sporozoite phenomena, suggesting that PRMT1 plays a crucial role in the dynamic changes of the centriole during the schizogony of the parasite [33]. These studies all indicate the importance of arginine methylation modification in the movement function and cell division of *T. gondii*. As the main type II PRMT responsible for SDMA, PRMT5 seems to be able to maintain the transcriptional program necessary for the stability of the cytoskeleton structure. Its deficiency would weaken the structural ability necessary for the parasite to achieve effective invasion and replication, which may be achieved through direct action on motor protein methylation or indirect action on the expression of genes related to the cytoskeleton.

Consistent with the in vitro phenotypes, the RH∆*prmt5* strain exhibited significantly attenuated virulence in the murine model, as evidenced by prolonged survival of infected mice and reduced parasite burden in vivo. This attenuation is likely multifactorial. On the one hand, the replication defect directly limits parasite expansion within the host. On the other hand, the downregulation of fatty acid oxidation pathways, particularly peroxisome and fatty acid degradation, suggests that *prmt5*-deficient parasites suffer from metabolic constraints. The tachyzoite stage of *T. gondii* exhibits high energy demands, and the fatty acid *β*-oxidation pathway is crucial for its growth and virulence through the degradation of stored long-chain and very-long-chain fatty acids and the detoxification of reactive oxygen species [34–36]. Although some studies have indicated that PRMT5 participates in the metabolism of bile acids, glucose, and lipids [37], the specific mechanisms by which PRMT5 influences metabolic functions remain poorly understood. Nevertheless, it is certain that TgPRMT5 deficiency leads to a significant reduction in both the proliferation capacity and virulence of *T. gondii*.

The conversion of *T. gondii* from tachyzoites to bradyzoites is a highly regulated process accompanied by extensive transcriptional reprogramming. Life cycle stage conversion relies on the precise regulation of gene expression through PTMs, which play a critical role in the developmental transition of the *T. gondii* [38]. This study found that under alkaline induction conditions, the expression level of the BAG1 gene in the RH∆*prmt5* strain was lower than that in the RH-WT strain. Concurrently, among the DEGs, BAG1 was also downregulated. Furthermore, 11 transcription factors from the AP2 transcription factor family (including (AP2X-8, TGME49-214960), (AP2IX-3, TGME49-264485), (AP2IX-9, TGME49-306620), (AP2IV-3, TGME49-318610), etc.) were found to be downregulated; while 3 transcription factors, (AP2VI-3, TGME49-244510), (AP2VIII-5, TGME49-271200), and (AP2X-6, TGME49-237425), were upregulated. Previous studies have shown that the AP2 transcription factor family is involved in regulating bradyzoite differentiation. For example, AP2IX-9 acts as a suppressor of bradyzoite differentiation, with its overexpression inhibiting tissue cyst formation and its deletion promoting stage conversion [39]. In contrast, AP2IV-3 functions as a transcriptional activator, enhancing tissue cyst formation when overexpressed [40]. The deletion of AP2IV-4 disrupts the normal regulation of bradyzoite genes [41]. AP2X-8 has been identified as a negative regulator of bradyzoite differentiation in *T. gondii* [42]. Additionally, one study investigating the proteomics of arginine monomethylation in *T. gondii* revealed that RNA-binding proteins and AP2 transcription factors are the primary types of proteins modified by MMA [15]. This study detected over ten AP2 transcription factors modified by arginine methylation, suggesting that MMA plays a significant role in the stage conversion of *T. gondii*. These findings imply that TgPRMT5 may regulate the tachyzoite-to-bradyzoite conversion process in *T. gondii* by methylating members of the AP2 transcription factor family. The specific underlying mechanism requires further experimental investigation.

Transcriptomic analysis provides a molecular framework for understanding the phenotypic abnormalities resulting from *prmt5* deficiency. Downregulated genes were almost exclusively enriched in pathways related to the cytoskeleton and fatty acid oxidation. Given that PRMT5-mediated methylation modifications on histones H4R3 and H3R8 are typically associated with transcriptional repression [43], we hypothesize that PRMT5-mediated methylation functions may impact the transcriptional stability of genes encoding cytoskeletal and metabolic proteins.

In summary, this study establishes PRMT5 as a crucial regulator in the biology of *T. gondii*, with its roles encompassing the functionality of motor proteins, metabolic homeostasis, and stage conversion. Through the analysis of phenotypic and transcriptomic data, we found that TgPRMT5 deletion leads to the downregulation of motility and fatty acid oxidation pathways, while concurrently upregulating pathways involved in proteasomal degradation and DNA replication and repair. Future research will focus on whether its function is abnormal after *prmt5* gene disruption and will further explore them by constructing complemented strains. Such investigations will not only deepen our understanding of the biological functions of arginine methyltransferase 5 in *T. gondii*, but may also reveal the potential of TgPRMT5 as a therapeutic target for toxoplasmosis.

## Conclusions

Our work has confirmed that TgPRMT5 plays a significant role in the virulence of *T. gondii*. Disrupting the *prmt5* gene leads to extensive transcriptional downregulation in *T. gondii*, particularly in genes involved in motor proteins and metabolic pathways and severe deficiencies in invasion, replication, virulence, and differentiation. These findings provide a deeper foundation for further functional research of TgPRMT5.

## Supplementary Information

Additional file 1.

Additional file 2.

Additional file 3.

Additional file 4.

## Data Availability

The RNA-seq datasets generated and analyzed in this study have been deposited in the NBCI Sequence Read Archive (SRA) under the BioProject accession PRJNA1457781. Other datasets supporting the findings of this article are included within the paper and its supplementary materials.

## Abbreviations

PRMT5: Protein arginine methyltransferase 5
SDMA: Symmetric dimethylarginine
*T. gondii*: *Toxoplasma gondii*
PTM: Post-translational modification
MMA: Monomethylarginines
ADMA: Asymmetric dimethylarginines
∆*prmt5*: *Prmt5* Disruption strain
PRMTs: Protein arginine methyltransferases
GFP: Green fluorescent protein
HFF: Human foreskin fibroblast cells
DMEM: Dulbecco’s modified Eagle’s medium
FBS: Fetal bovine serum
PFA: Paraformaldehyde
BSA: Bovine serum albumin
PV: Parasitophorous vacuole
qPCR: Quantitative real-time polymerase chain reaction
RNA-seq: RNA sequencing
DEGs: Differentially expressed genes
KEGG: Kyoto Encyclopedia of Genes and Genomes
GO: Gene Ontology
SPF: Specific Pathogen-Free
RH: RH strain of Toxoplasma gondii
eGFP: enhanced Green Fluorescent Protein
DHFRTs: Dihydrofolate Reductase-Thymidylate Synthase, DHFR-TS
SAM: S-adenosylmethionine
PBS: Phosphate-Buffered Saline
RIPA: Radio-Immunoprecipitation Assay
TBST: Tris-Buffered Saline with Tween 20
HRP: Horseradish Peroxidase
ECL: Enhanced Chemiluminescence
HEPES: 4-(2-hydroxyethyl)-1-piperazineethanesulfonic acid
SDS-PAGE: Sodium Dodecyl Sulfate–Polyacrylamide Gel Electrophoresis
TPM: Transcripts Per Million
