# Supplementary material for: Protein arginine methyltransferase 5 is essential for virulence in Toxoplasma gondii
Source: Parasit Vectors. 2026 Jun 2;19:317. doi: 10.1186/s13071-026-07473-3 (PMC13445752; doi:10.1186/s13071-026-07473-3)
Supplement: Supplementary file 1 [file 13071_2026_7473_MOESM3_ESM.docx]

**Supplementary Figure**

**Figure. S1 (A)** Correlation coefficient between RH-WT strain and RH∆*prmt5* strain samples. **(B)** The TPM density distribution map of the RH-WT strain and the RH∆*prmt5* strain genes.

**Figure. S2 A)** The GO Term of up-regulation of DEGs. **B)** The GO Term of down-regulation of DEGs. **C)** KEGG enrichment bubble plot showing the upregulation of DEGs. **D)** KEGG enrichment bubble plot showing the downregulation of DEGs.

**Figure. S3** Typical photographs of RH∆prmt5 viewed at 400 magnification

**Figure. S4** The expression of GFP protein in RH∆*prmt5* was detected (before cropping)**.**

**Figure.S5** Invasion experiment. Images of RH-GFP were taken at 400× and images of RH∆*prmt5* were taken at 1000×.

**Figure S6**. SDMA levels in RH-WT and RH∆*prmt5* strains detected by western blot
