# Supplementary material for: Protein arginine methyltransferase 5 is essential for virulence in Toxoplasma gondii
Source: Parasit Vectors. 2026 Jun 2;19:317. doi: 10.1186/s13071-026-07473-3 (PMC13445752; doi:10.1186/s13071-026-07473-3)
Supplement: Supplementary file 2 [file 13071_2026_7473_MOESM4_ESM.docx]

**Supplementary Table 1. Primers used for the construction of *prmt5* disruption strain**

| Name of primers | Primer sequences |
| --- | --- |
| gRNA-Fw(PRMT5) | GCTGGAGAAAGCGCGCGACGGTTTTAGAGCTAGAAATAGC |
| gRNA-Rv (3’-ROP18-gRNA) | AACTTGACATCCCCATTTAC |
| PRMT5-HR-up-F | GCGGGCCCCGCTTCGTTTGCGTCTTGT |
| PRMT5-HR-up-R | CGGGATCCGCGACAGATACGGCTGGAGAT |
| PRMT5-HR-down-F | ATAAGAATGCGGCCGCGACAACCTAAGCACGATGAACT |
| PRMT5-HR-down-R | CTGCAGAACCACCGCGGTGGCGTCGTCAGCGAAAGGGA |
| eGFP-F | CGGGATCCGTGAGCAAGGGCG |
| eGFP-R | GACTAGTCTCGGGGGGGCAAGAATTG |
| DHFRTs-F | GCTCTAGAAAGCTTCGCCAGGCTGTAAATC |
| DHFRTs-R | ATAAGAATGCGGCCGCGGAATTCATCCTGCAAGTGCATAGAAG |
| Check-PCR1-F1 | TCAGTCCGAGACACTTTGCC |
| Check-PCR1-R1 | TTCGCCTTGATGCCGTTC |
| Check-PCR2-F2 | GCCTTGTCACTTGTTGTGC |
| Check-PCR2-R2 | GCGGCTCTTCTTCTTGGA |
| Check-PCR3-F3 | GGCAGCAGTGGAGGTTGAT |
| Check-PCR3-R3 | GGACGTACTTGACAGGGTCTTT |

**Supplementary Table 2. Primers used for qPCR**

| Name of primers | Primer sequences |
| --- | --- |
| TGME49_226540-F | TCGCGCTCCTGAGATGTACT |
| TGME49_226540-R | CTGCCTCTTCCACCGAACAA |
| TGME49_216450-F | CGCATTGGGCTCTTCATGC |
| TGME49_216450-R | GCAGTAGAGCTCGCTCTTGAA |
| TGME49_201850-F | GAGGGCCAGAGTCATACAGC |
| TGME49_201850-R | ATACCAAACAACTCCGCCGT |
| TGME49_310830-F | ATGCACTTGGTCGCTCTCAA |
| TGME49_310830-R | TTTCTCCTGAACCCGTGTCG |
| TGME49_248330-F | CCGGATCACCAGTGCAAAAG |
| TGME49_248330-R | GAAGATCGCAAGGTGAAGCC |
| TGME49_247500-F | GTTGGCGGAGGAGGTTGTTA |
| TGME49_247500-R | ACCCAGTAACGTGCCTGTTC |
| TGME49_273740-F | CGAAGGCAAAAAGAGGTGGC |
| TGME49_273740-R | CCATAAGCATGCCAATCCGC |
| TGME49_231900-F | TGGCTCCCTAAGATGTCACG |
| TGME49_231900-R | CAGACAACCACGATGTCCGA |
| TGME49_315480-F | CGTCGAGCAATTTAGGGAGC |
| TGME49_315480-R | AAGCAAATGAGAGCGTGGGA |
| TGME49_221320-F | TGCCCTCCTACAAAGTTGGC |
| TGME49_221320-R | TGAAGGCCTCAGCCTTGAAC |
| TGME49_258880-F | CGACCAACACTGGTCCGATA |
| TGME49_258880-R | CCCACGTCAAGGTAAGTCGT |
| TGME49_263030-F | TTCCGAAATGCACGGAGACG |
| TGME49_263030-R | ACAGTGCCAGTTTGGTGAGT |
| TGME49_245670-F | GAGATGAGGGCAGTCAAGCA |
| TGME49_245670-R | CGACCACAGCTTTCACTTTCG |
| TGME49_268850-F | TGCTCGTCAGATCCTCGACTC |
| TGME49_268850-R | ATCACACCCTTGCCCATGAA |
| TGME49_281510-F | CTGATCCTGCGAACGGTGAA |
| TGME49_281510-R | CCTTCTCCGCCACCTTGATTT |
| SAG1-F | GCCTCATCGGTCGTCAATAA |
| SAG1-R | GTCATTGTAGTGGGTCCTTCC |
| BAG1-F | CCTCGAAAGAAGCGGAGAAA |
| BAG1-R | GATTCCGTCGGGCTTGTAAT |
| B1-F | GGAGGACTGGCAACCTGGTGTCG |
| B1-R | TTGTTTCACCCGGACCGTTTAGCAG |
| β-tubulin-F | ATGTTCCGTGGTCGCATGT |
| β-tubulin-R | TGGGAATCCACTGAACGAAGT |

**Supplementary Table 3. Summary of sample sequencing data quality**

| Samples | Raw reads | Clean reads | GC (%) | Q20(%) | Q30(%) | Error rate (%) |
| --- | --- | --- | --- | --- | --- | --- |
| RH∆*prmt5*① | 30165127 | 29402200 | 52.04 | 98.65 | 95.54 | 0.25 |
| RH∆*prmt5*② | 30308372 | 29529482 | 53.67 | 98.65 | 95.57 | 0.25 |
| RH∆*prmt5*③ | 29237704 | 28550949 | 52.78 | 98.62 | 95.49 | 0.24 |
| RH-WT① | 22705091 | 22220960 | 53.63 | 98.70 | 95.71 | 0.24 |
| RH-WT② | 30597713 | 29884104 | 53.71 | 98.66 | 95.62 | 0.25 |
| RH-WT③ | 22773987 | 22190577 | 52.86 | 98.68 | 95.62 | 0.24 |

**Supplementary Table 4. Information of 15 DEGs**

|  | Gene | Regulation | Description | KEGG Pathway |
| --- | --- | --- | --- | --- |
| 1 | TGME49_226540 | down | protein kinase | Toxoplasmosis |
| 2 | TGME49_216450 | up | peptidase, T1 family protein | Proteasome |
| 3 | TGME49_201850 | up | WD domain, G-beta repeat-containing protein | Peroxisome |
| 4 | TGME49_310830 | down | enoyl-CoA hydratase/isomerase family protein | Peroxisome |
| 5 | TGME49_248330 | down | zinc finger, C3HC4 type (RING finger) domain-containing protein | Peroxisome |
| 6 | TGME49_247500 | down | acyl-CoA dehydrogenase, middle domain-containing protein | Peroxisome/  Fatty acid metabolism |
| 7 | TGME49_273740 | down | acetyl-CoA acyltransferase B, putative | Peroxisome/  Fatty acid metabolism |
| 8 | TGME49_231900 | down | acyl-CoA dehydrogenase domain-containing protein | Peroxisome/  Fatty acid metabolism |
| 9 | TGME49_315480 | down | acyl-CoA dehydrogenase, middle domain-containing protein | Peroxisome/  Fatty acid metabolism |
| 10 | TGME49_221320 | up | acetyl-CoA carboxylase ACC1 | Fatty acid metabolism/  Metabolic pathways |
| 11 | TGME49_258880 | down | axonemal dynein light chain p33, putative | Motor proteins |
| 12 | TGME49_263030 | down | dynein light chain 8 family E protein | Motor proteins |
| 13 | TGME49_245670 | up | pyruvate dehydrogenase complex subunit PDH-E1Alpha | Glycolysis / Gluconeogenesis\| Citrate cycle (TCA cycle) \|Pyruvate metabolism |
| 14 | TGME49_268850 | up | enolase 2 | Glycolysis / Gluconeogenesis |
| 15 | TGME49_281510 | up | ribonuclease H1 large subunit, putative | DNA replication |
| 16 | TGME49_203050 | down | AP2 domain transcription factor AP2VIIa-6 |  |
| 17 | TGME49_208020 | down | AP2 domain transcription factor AP2Ib-1 |  |
| 18 | TGME49_244510 | up | AP2 domain transcription factor AP2VI-3 |  |
| 19 | TGME49_214960 | down | AP2 domain transcription factor AP2X-8 |  |
| 20 | TGME49_269010 | down | AP2 domain transcription factor AP2VIII-7 |  |
| 21 | TGME49_272710 | down | AP2 domain transcription factor AP2VIII-4 |  |
| 22 | TGME49_264485 | down | AP2 domain transcription factor AP2IX-3 |  |
| 23 | TGME49_306620 | down | AP2 domain transcription factor AP2IX-9 |  |
| 24 | TGME49_271200 | up | AP2 domain transcription factor AP2VIII-5 |  |
| 25 | TGME49_237425 | up | AP2 domain transcription factor AP2X-6 |  |
| 26 | TGME49_215340 | down | AP2 domain transcription factor AP2X-10 |  |
| 27 | TGME49_318610 | down | AP2 domain transcription factor AP2IV-3 |  |
| 28 | TGME49_252370 | down | AP2 domain transcription factor AP2III-1 |  |
| 29 | TGME49_299020 | down | AP2 domain transcription factor AP2III-4 |  |
| 30 | TGME49_246660 | down | AP2 domain transcription factor AP2XII-3 |  |
